# Association of cancer screening and residing in a coal-polluted East Asian region with overall survival of lung cancer patients: a retrospective cohort study

**DOI:** 10.1038/s41598-020-74082-0

**Published:** 2020-10-15

**Authors:** Runxiang Yang, Ming He, Dongmei Wang, Rongrong Ye, Lu Li, Rouyu Deng, Mohsin Shah, Sai-Ching Jim Yeung

**Affiliations:** 1 The Second Department of Medical Oncology, Yunnan Cancer Hospital, Kunming, Yunnan The People’s Republic of China; 2 Department of Oncology, Sun Yat-Sen University Cancer Center, Guangzhou, Guangdong The People’s Republic of China; 3 Center for Clinical Epidemiology and Biostatistics, Perelman School of Medicine, University of Pennsylvania, Philadelphia, PA USA; 4 Department of Emergency Medicine, The University of Texas MD Anderson Cancer Center, Houston, TX USA

**Keywords:** Cancer epidemiology, Lung cancer

## Abstract

Lung cancer is the leading cause of cancer death worldwide. The Xuanwei-Fuyuan (XF) region of Yunnan, China has a high incidence of lung cancer from coal-related pollution. Effort to raise public awareness screening for lung cancer has been ongoing. We retrospectively analyzed overall survival (OS) of lung cancer patients of a tertiary cancer center in Yunnan to investigate screening and regional residential status as predictive factors. Consecutive cases of newly diagnosed lung cancer were reviewed. The lung cancer cases diagnosed by screening were more likely to be early-staged and treated by surgery than those diagnosed not by screening. In patients diagnosed not by screening, XF residential status was a significant predictor of improved OS. Frailty model detected significant heterogeneity associated with region of residence in unscreened patients. Potential biases associated with screening were examined by Monte Carlo simulations and sensitivity analyses. Focused effort in cancer screening and increased public awareness of pollution-related lung cancer in XF might have led to early diagnosis and improved OS, and increased investment in health care resources in high risk areas may have produced additional unobserved factors that underlay the association of XF residential status with improved OS in patients diagnosed not by screening.

## Introduction

Lung cancer remains the leading cause of cancer mortality worldwide with an estimated 1.4 million deaths annually and a 5-year survival of 17%^1,2^. It accounts for 20% of cancer-related deaths in America and ranks first in incidence and mortality rates for both males and females in China^3^. Tobacco smoking is the primary risk factor for developing lung cancer; other factors include occupational exposures (e.g. asbestos, silica and chromium), environmental tobacco smoke, indoor coal/wood emissions, radon, family history, and pulmonary fibrosis^4–6^. Particularly, there is an increased prevalence of adenocarcinoma in Asian non-smokers, especially females, compared with western countries^6^. Geographic patterns in cancer occurrence provide clues to the role of environmental or lifestyle factors affecting cancer risk^7^. Moreover, population-based cancer survival is an important index that assists in evaluating the overall efficiency of cancer health services in a region^7^.

Yunnan, a southwestern province of China, has a lung cancer incidence rate which is twice that of the whole China^2^. In Yunnan, Xuanwei and Fuyuan (XF) are two neighboring counties where coal burning is ubiquitous (infamily cooking, heating and industrial production, etc.). Polycyclic aromatic hydrocarbons and other suspended particles (e.g., nano-quartz) produced by bituminous coal combustion lead to serious air pollution^3,8^. Environmental pollution is perhaps the most important factor in lung carcinogenesis in XF residents^9^. Since the 1970s, various governmental efforts tried to combat pollution-related lung cancer in XF residents. The change from unvented fire pits to stoves with chimneys reduced indoor air pollution by more than 65%, and was associated with reduction in lung cancer incidence 10 years after the change^10^. In addition to environmental measures, governmental efforts to strengthen the lung cancer screening, early diagnosis and early treatment were undertaken^11,12^. Although low-dose computed tomography (CT) lung screening may decrease lung cancer mortality by diagnosing in early stages^13^, the efficiency of lung cancer screening may depend on the selection of patients for screening^14^ and the criteria used in the interpretation of radiological images^15,16^. In spite of screening efforts, the mortality rates in Xuanwei based on the data from 2004 to 2005^17^ and those from 2011 to 2015^18^ were similar. Improvement in early diagnosis and lung cancer mortality was expected to lag behind sustained public health efforts of lung cancer screening. We hypothesized that lung cancer cases diagnosed by screening would be diagnosed at earlier stages and have better prognosis than cases diagnosed not by screening.

Retrospective analysis of cohorts in which screening have taken place is complicated by surveillance (detection) bias, lead time bias and length bias. Surveillance bias primarily affect the analysis of cancer risk, and is difficult to mitigate in retrospective observational studies^19^. The estimated mean lead time for overall survival of lung cancer is 3.4 months for stages I and II and ≤ 1 month for stages III and IV^20^. We investigated whether cancer screening is still a significant factor for improved overall survival after correction for length bias or lead time bias.

## Material and methods

### Participants, study and design

The study was approved by the Yunnan Cancer Hospital Institutional Review Board, and was carried out according to the research protocol in compliance with the Declaration of Helsinki**.** Yunnan Cancer Hospital is the provincial cancer center and the tertiary referral center for cancer patients in Yunnan province^21^, and the patient population comes from and is representative of all over Yunnan. The cohort for the current study consisted of consecutive new cases of lung cancer patients at Yunnan Cancer Hospital from January 1, 2012 to July 10, 2015. Participants were excluded from the study: (i) if they were not residents of Yunnan, (ii) carcinoma in situ (stage 0), or (iii) had received cancer treatments (radiation, chemotherapy or surgery) at other hospitals. A total of 3859 cases were then reviewed and proceeded to telephone follow up. The informed consent for telephone follow up was obtained verbally from all subjects with successful follow up because obtaining a written consent was not feasible during telephone follow up, and this was approved by the Institutional Review Board. All participants were over the age of 19 years.

### Data variables

Each address was confirmed, and the classification whether it belonged to XF was based on the postal code, and the geographic location of residence at the time of diagnosis was confirmed by plotting the latitude and longitude of each address (obtained using “Geocode”) on a map. Clinical chart review and data abstraction were performed by experienced oncologists. Demographic (ethnicity (Han vs. non-Han), gender, age at diagnosis), Clinicopathological characteristics of lung cancer, cancer stage based on the American Joint Committee on Cancer (AJCC) TNM staging system, cancer treatments, body mass index (BMI), Karnofsky performance score (KPS), comorbidities and clinic follow up information were abstracted from clinical records. BMI was categorized for analysis according to Asian-Pacific recommendations^22^. Comorbidity information was used for calculating the age-unadjusted Charlson Comorbidity Index (CCI). Data were also collected about the presenting symptoms (i.e., fever, cough, sputum, blood-tinged sputum, hemoptysis, chest pain, chest pressure, dyspnea on exertion, dyspnea at rest, skeletal symptoms, gastrointestinal symptoms, and neurological symptoms), and symptoms deemed relevant to lung cancer by the chart reviewers were recorded. The group of patients diagnosed by screening was defined as the asymptomatic patients who were discovered to have pulmonary lesions in chest radiographs or CT scan of the chest during routine checkup visits or cancer screening. Clinical outcome was obtained by telephone follow up to obtain the date of death from surviving family members, and for cases who were still alive, they were censored at the date of telephone follow up. There were 454 (11.7%) patients that did not have a successful telephone follow up.

### Statistical analysis

Descriptive statistics were presented as frequencies and percentages for the categorical variables. Student’s t test or nonparametric rank sum test was used to compare two groups where appropriate. Ratios and proportions were compared by Pearson Chi-square test. Kaplan–Meier method was used to analyze overall survival (OS) rates with log-rank test to determine statistical differences between groups. Random survival forest (R package “ranger”) was performed to evaluate the relative importance of various factors in predicting OS. Multivariate Cox proportional hazard models were chosen a priori with consideration of results from random survival forest. Multicollinearity was assessed by the variance inflation factor (VIF) of each covariate. Hazard ratios (HRs) were reported with their 95% confidence intervals (CIs). The Cox models were validated by examining scaled Schoenfeld residuals and Martingale residuals (R package “ggcoxdiagnostics”). Latent factors affecting OS were assessed using shared frailty models (R package “survival”). Sensitivity analysis of Cox regression model was performed using the R package “obsSens”.

Correction of relative risk of death for length time bias and correction of survival time for lead time were based on the method by Duffy et al.^23^ that assumed an exponentially distributed sojourn time^24^. For each scenario of a specified mean rate of transition to symptomatic disease λ and its standard deviation, the observed overall survival time of each screen-detected lung cancer case was corrected by subtracting a lead time [*E*(*s*)] calculated using the published formula [*E*(*s*) = (1−e^-λt^)/λ]^23^, and randomized λ for the screen-detected cases (using the rnorm() function in R) for the specified mean and standard deviation (SD) of λ, Cox regression analysis of each data set after correction of lead time for screened cases was performed. The probability of obtaining P > 0.05 for the association of screening and XF residential status with OS out of 1000 randomizations were calculated for each combination of mean and SD of λ. The estimated true relative hazard after correcting for length time bias (Ψ) was calculated using formulae by Duffy et al.^23^, with the following assumptions: (1) the survival function is exponential^24^; (2) there exists a non-aggressive cancer subtype that is more likely to be screen-detected than the rest of the cancers. In the calculation of estimated true relative risk of death independent of length bias for screen-diagnosed cases compared to symptomatic cases (φ), p1 was assumed to be the observed 5-year case fatality for symptomatic cancers, p2 was assumed to be the observed 5-year case fatality for screen-detected lung cancers, and p3 was the observed probability of screen detection in the entire cohort. Using the above assumed values for p1, p2 and p3, and Ψ was calculated for random combinations of values (between 0 and 1) of the ratio of probability of death from aggressive cancer subtype to that from the non-aggressive cancer subtype (θ) and the proportion of aggressive cancer subtype in the whole cohort (q). The R package “MonteCarlo” was used to perform the calculations.

Statistical analysis was performed using Statistical Package for the Social Sciences (SPSS) version 20.0 (IBM Analytics, USA) and R statistical software (version 3.6.2, R Foundation for Statistical Computing, Vienna, Austria. https://www.R-project.org/). Except when Bonferroni correction for multiple testing was applied, *P* < 0.05 was considered statistically significant.

### Ethical approval

Institutional IRB of the Yunnan Cancer Hospital.

## Results

### Demographic and Clinicopathological Characteristics

Between 1/1/2012 and 7/10/2015, 7740 consecutive admissions of newly discovered lung masses or newly diagnosed lung cancer were identified from the hospital database. After applying the aforementioned exclusion criteria, 3859 cases were reviewed and followed up by telephone (Supplemental Fig. 1). The number of patients from XF or non-XF regions and their ratios did not vary a lot over the years of the study (Supplemental Table 1). Among these, 3405 reviewed cases of newly diagnosed lung cancer with successful telephone follow up were analyzed. There were 13.9% of patients in the XF group, who were unable to be contacted by telephone or refused to participate in the study follow up, and 12.6% of patients such patients in the non-XF group. The rates of unsuccessful telephone follow up were very similar for both groups.

**Table 1:** Demographic and clinicopathological characteristics.

	Diagnosed by Screening	No	Yes	No	Yes	
	XF residents	No	No	Yes	Yes	
N (total)		2565	143	574	123	
Variables	Level					p
Age		58.63 ± 10.74	57.31 ± 10.77	53.62 ± 10.15	50.11 ± 8.99	< 0.001
Sex	Female	708 (27.6)	65 (45.5)	218 (38.0)	52 (42.3)	< 0.001
	Male	1857 (72.4)	78 (54.5)	356 (62.0)	71 (57.7)	
Ethnicity	Han	2364 (92.2)	137 (95.8)	566 (98.6)	122 (99.2)	< 0.001
	Non-Han	201 (7.8)	6 (4.2)	8 (1.4)	1 (0.8)	
TNM stage	I	192 (7.5)	63 (44.7)	119 (20.8)	55 (44.7)	< 0.001
	II	188 (7.3)	14 (9.9)	69 (12.0)	25 (20.3)	
	III	799 (31.2)	36 (25.5)	155 (27.1)	27 (22.0)	
	IV	1380 (53.9)	28 (19.9)	230 (40.1)	16 (13.0)	
Cancer histology	Adenocarcinoma	1017 (39.6)	105 (73.4)	342 (59.6)	103 (83.7)	< 0.001
	Others	466 (18.2)	18 (12.6)	100 (17.4)	12 (9.8)	
	Small Cell	341 (13.3)	5 (3.5)	46 (8.0)	3 (2.4)	
	Squamous Cell	741 (28.9)	15 (10.5)	86 (15.0)	5 (4.1)	
BMI categories^#^	< 18.5	307 (12.2)	6 (4.2)	55 (9.6)	4 (3.3)	0.004
	≥ 18.5 & < 23	1311 (51.9)	70 (49.0)	293 (51.4)	66 (53.7)	
	≥ 23 & < 25	478 (18.9)	32 (22.4)	115 (20.2)	28 (22.8)	
	≥ 25	430 (17.0)	35 (24.5)	107 (18.8)	25 (20.3)	
Age-unadjusted CCI		6 [2, 12]	2 [2, 9]	2.00 [2, 8]	2 [2, 7]	< 0.001
KPS		80[30, 100]	100[80, 100]	90[50, 100]	100 [80, 100]	< 0.001
Smoking	No	1247 (48.6)	99 (69.2)	306 (53.3)	74 (60.2)	< 0.001
	Yes	1318 (51.4)	44 (30.8)	268 (46.7)	49 (39.8)	
Surgery	No	1958 (76.3)	33 (23.1)	292 (50.9)	17 (13.8)	< 0.001
	Yes	607 (23.7)	110 (76.9)	282 (49.1)	106 (86.2)	
Chemotherapy	No	963 (37.5)	49 (34.3)	233 (40.6)	54 (43.9)	0.213
	Yes	1602 (62.5)	94 (65.7)	341 (59.4)	69 (56.1)	
Radiotherapy	No	2138 (83.4)	126 (88.1)	497 (86.6)	115 (93.5)	0.004
	Yes	427 (16.6)	17 (11.9)	77 (13.4)	8 (6.5)	
Targeted therapy	No	2276 (88.7)	131 (91.6)	542 (94.4)	116 (94.3)	< 0.001
	Yes	289 (11.3)	12 (8.4)	32 (5.6)	7 (5.7)	

Continuous variables are reported as means ± standard deviations; counts are reported along with percentages in parentheses; non-normally distributed values are reported as modes along with the ranges in square brackets.

*BMI* body-mass index, *CCI* Charlson Comorbidity Index, *KPS* Karnofsky Performance Score, *TNM* Tumor-Lymph Node-Metastasis, *XF* Xuanwei–Fuyuan.

*After Bonferroni correction for multiple testing, a *P* value < 0.00366 will be statistically significant.

^#^Asian-Pacific BMI classification: underweight (BMI < 18.5), normal (18.5 ≥ BMI < 23), overweight (23 ≥ BMI < 25), obese (BMI ≥ 25).

**Figure 1:**
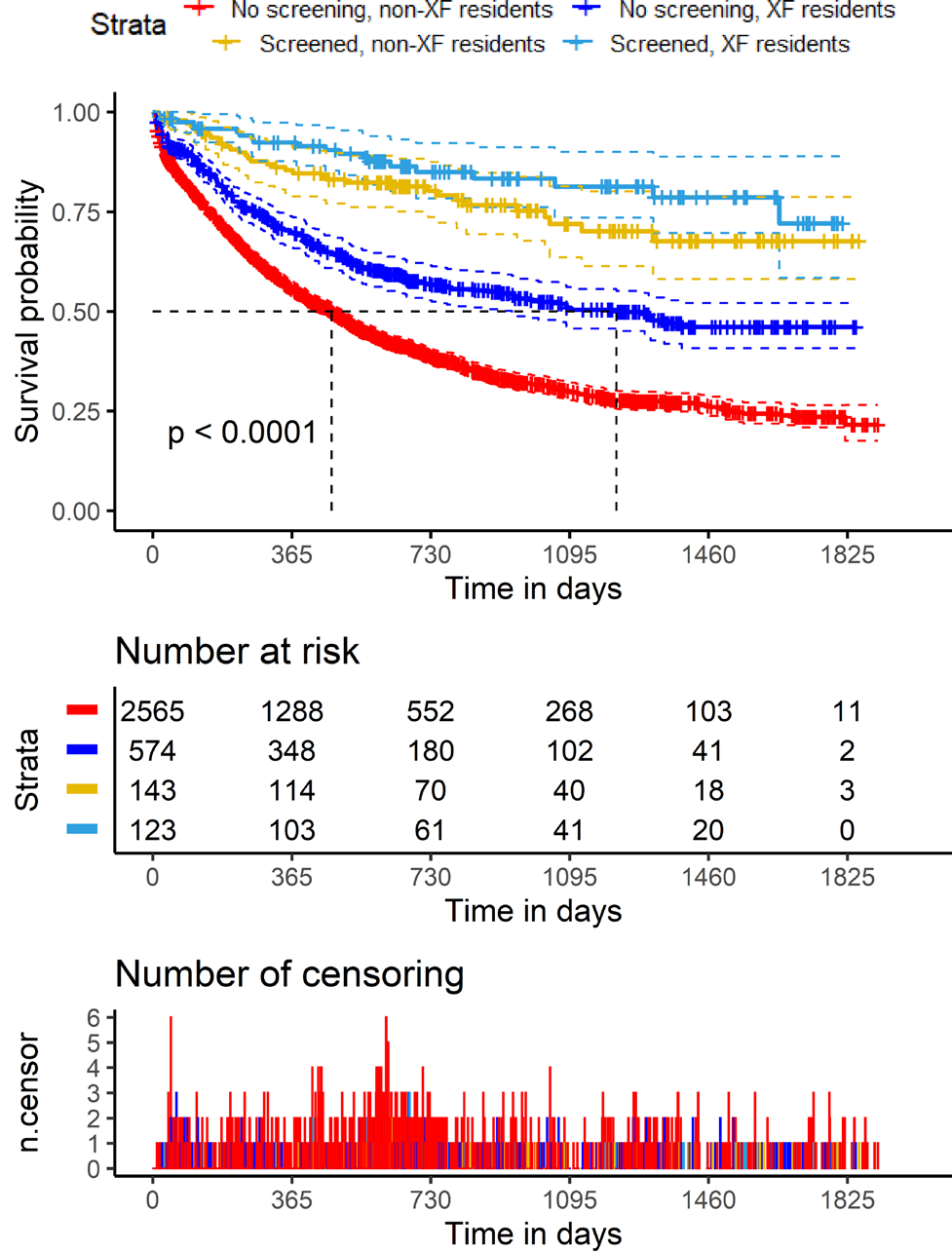
Kaplan–Meier analysis of overall survival of lung cancer patients. The study cohort was divided into strata based on residency in Xuanwei-Fuyuan (XF) and whether the lung cancer was diagnosed by screening (see color key). For patients diagnosed not by screening, XF residents (blue) had better prognosis than the residents of the rest of Yunnan Province (red) (top panel; *P* < 0.001, log rank test). The dashed lines above and below the survival curves mark the 95% confidence intervals. The number of patients at risk were tabulated (middle panel), and the number of patients censored were plotted against time (bottom panel).

There were 266 (7.8%) cases diagnosed by lung cancer screening (123 in XF residents and 143 in non-XF residents). There were 698 cases (20.4%) from XF. The longitude and latitude of the addresses of XF patients were verified by plotting the address of residence of each case on the map (Supplemental Fig. 2). The demographic and clinicopathological characteristics were summarized in Table 1.

**Figure 2:**
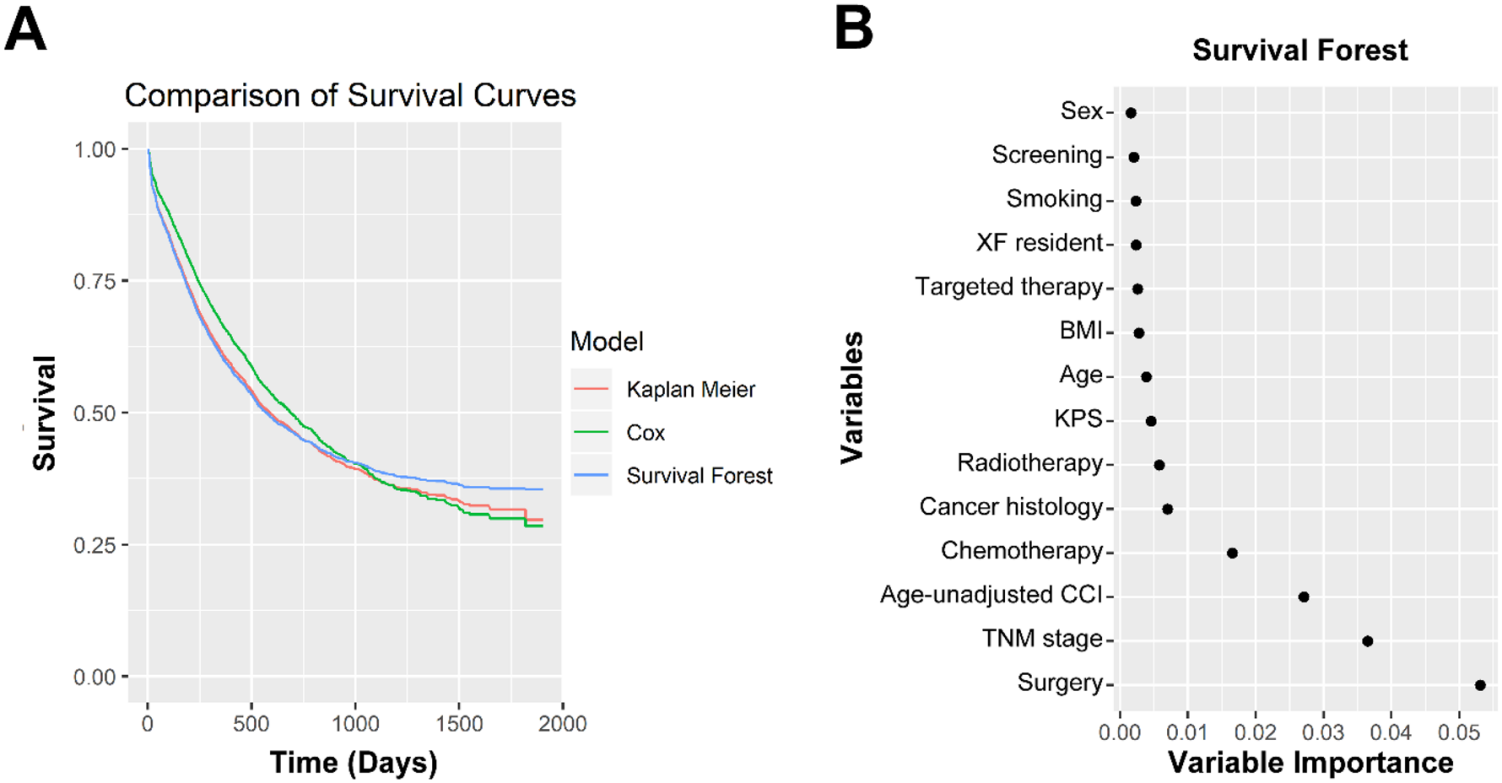
Random survival forest results. (**A**) Survival curves estimated using three methods were plotted for comparison. Red: Kaplan–Meier method; green: Cox proportional hazard regression; blue: random survival forest. (**B**) Plot of the top 14 variables with the highest variable importance in random survival forest analysis.

Patients diagnosed by screening accounted for 27.4% of all stage I patients, and 46.6% of stage I patients diagnosed by screening resided in XF. Almost all (98.4%) of stage I patients underwent surgical resection of the primary cancer. In contrast, the majority of the patients not diagnosed through screening had stage IV cancer.

The male:female ratio in non-XF patients diagnosed not by screening was 2.62:1 while that ratio in XF patients diagnosed by screening was 1.37:1. The percentage of XF patients diagnosed by screening was 17.6%, which was higher than non-XF patients (5.3%; *P* < 0.001). The distributions of histopathological types were different (*P* < 0.001) between the two regions with adenocarcinoma accounting for 63.9% of XF cases. A higher percentage of patients diagnosed by screening were diagnosed in stage I and II (XF: 65%, non-XF: 54.6%) than patients diagnosed not through screening (XF: 32.7%, non-XF: 14.8%) (*P* < 0.001). Concordantly, there were higher percentages of patients treated with surgery in patients diagnosed by screening and XF patients than patients diagnosed not by screening and non-XF patients (*P* < 0.001).

In summary, patients diagnosed by screening were more likely to be diagnosed in early stages (I and II). There were more female patients with adenocarcinoma of the lung in XF than other parts of Yunnan. XF lung cancer patients were more likely to be diagnosed by screening and were diagnosed at earlier stages than non-XF patients. The patients diagnosed by screening were diagnosed at younger ages, had less comorbidity and were more likely to undergo surgical treatment than patients diagnosed not by screening.

### Survival analyses

#### Examination of screening and XF residential status as predictive factors of OS.

We first examined whether there were differences in OS associated with lung cancer screening and XF residency status. Kaplan–Meier analysis showed that the OSs of screened patients (median not reached for both XF and non-XF patients) were higher than those of patients not diagnosed by screening (unscreened non-XF patients: median OS = 463 days, unscreened XF patients: median OS = 1218 days) (Fig. 1, log rank test: *P* < 0.001). The 5-year survival rates were 72.7% for screened XF patients, 66.3% for screened non-XF patients, 46.2% for unscreened XF patients, 22.5% for unscreened non-XF patients. A random forest machine learning strategy (“ranger” R package) was used to identify important covariates among a total of 49 (including demographic and social characteristics, clinicopathological characteristics, cancer treatment modalities, screening, presenting symptoms and comorbidities)^25^. The random survival forest method obviates imposition of semi-parametric or parametric constraints and automatically addresses interactions among variables to predict survival accurately^26^. The Kaplan–Meier, Cox proportional hazard and random survival forest methods produce very similar models of OS (Fig. 2A), and XF residential status was among the top twelve predictive factors based on the “relative importance” values calculated in the random forest method (Fig. 2B).

#### Cox proportional hazard regression analysis

Given the above findings about the differences between diagnosis by screening and geographic regions of residence (XF vs. non-XF) patients in terms of lung cancer histology, age at diagnosis, CCI and stage of cancer, it was clear that XF residents were more likely to have lung cancer screening and were diagnosed at younger ages, had less comorbidity and were more likely to undergo surgical treatment than non-XF patients. In addition to residency status and screening, we used univariate Cox proportional hazard analysis for the following variables: diagnosis by screening (yes vs. no), XF residency (yes vs. no), demographic factors (age > 65 years, sex, ethnicity, smoking), clinicopathological factors (age-unadjusted CCI > 3, KPS > 70, BMI categories for the Asian and Pacific population, surgery, radiotherapy, chemotherapy, targeted therapy, cancer histology, and TNM stage) (Table 2). To test the hypothesis that XF residency status and screening were independent predictors of OS, we examined a multivariate Cox regression model constructed a priori with these variables. Age at diagnosis > 65 years, male sex, non-Han ethnicity, higher cancer stages were associated with poor OS and KPS > 70, surgery, radiotherapy, chemotherapy, targeted therapy, diagnosis by screening and XF residency were associated with improved OS (Table 2). Proportional hazard assumption was checked as described in Methods and was not violated (data not shown). Therefore, XF residency status and screening both appeared to be independent predictors of improved OS.

**Table 2:** Univariate and multivariate Cox proportional hazard analysis for predictors of overall survival.

Factor	Level	Univariate Analysis				Multivariate Model			
		beta	se	HR (95% CI)	p	beta	se	HR (95% CI)	p
Age (Ref: ≤ 65 years)	> 65 years	0.414	0.0506	1.51 (1.37–1.67)	< 0.001	0.131	0.0536	1.14 (1.03–1.27)	0.0145
Sex (Ref: female)	Male	0.336	0.0524	1.4 (1.26–1.55)	< 0.001	0.189	0.0708	1.21 (1.05–1.39)	0.0076
Ethnicity (Ref: non-Han)	Han	0.318	0.0873	1.37 (1.16–1.63)	< 0.001	0.228	0.0904	1.26 (1.05–1.5)	0.0117
TNM stage (Ref: stage I)	Stage II	1.03	0.169	2.8 (2.01–3.9)	< 0.001	0.959	0.173	2.61 (1.86–3.66)	< 0.001
	Stage III	1.82	0.14	6.2 (4.71–8.16)	< 0.001	1.4	0.151	4.06 (3.02–5.45)	< 0.001
	Stage IV	2.37	0.137	10.7 (8.19–14)	< 0.001	1.88	0.283	6.55 (3.77–11.4)	< 0.001
Cancer histology (Ref: adenocarcinoma)	Others	0.815	0.0621	2.26 (2–2.55)	< 0.001	0.14	0.0699	1.15 (1–1.32)	0.0452
	Small cell	0.713	0.0714	2.04 (1.77–2.35)	< 0.001	0.11	0.0795	1.12 (0.955–1.3)	0.166
	Squamous cell	0.323	0.0591	1.38 (1.23–1.55)	< 0.001	0.0171	0.0696	1.02 (0.887–1.17)	0.806
BMI^#^ (Ref: normal)	Obese	− 0.332	0.0681	0.717 (0.628–0.82)	< 0.001	− 0.162	0.0692	0.85 (0.743–0.974)	< 0.001
	Overweight	0.315	0.072	1.37 (1.19–1.58)	< 0.001	0.0924	0.0727	1.1 (0.951–1.26)	0.204
	Underweight	− 0.148	0.0631	0.862 (0.762–0.976)	0.0187	− 0.0197	0.0636	0.98 (0.866–1.11)	0.757
Age-unadjusted CCI (Ref: ≤ 3)	> 3	0.962	0.0481	2.62 (2.38–2.88)	< 0.001	− 0.111	0.246	0.895 (0.552–1.45)	0.652
KPS (Ref: ≤ 70)	> 70	− 0.747	0.0655	0.474 (0.417–0.538)	< 0.001	− 0.219	0.0703	0.803 (0.7–0.922)	0.00184
Smoking (Ref: no)	Yes	0.285	0.0463	1.33 (1.21–1.46)	< 0.001	0.107	0.0614	1.11 (0.987–1.26)	0.0814
Surgery (Ref: no)	Yes	− 1.51	0.0647	0.22 (0.194–0.25)	< 0.001	− 0.731	0.0781	0.481 (0.413–0.561)	< 0.001
Chemotherapy (Ref: no)	Yes	− 0.482	0.0466	0.618 (0.564–0.677)	< 0.001	− 0.413	0.0561	0.662 (0.593–0.738)	< 0.001
Radiotherapy (Ref: no)	Yes	− 0.186	0.0628	0.83 (0.734–0.939)	0.00304	− 0.244	0.068	0.783 (0.686–0.895)	< 0.001
Targeted therapy (Ref: no)	Yes	− 0.168	0.0775	0.846 (0.727–0.984)	0.0305	− 0.313	0.0831	0.731 (0.621–0.86)	< 0.001
XF resident (Ref: no)	Yes	− 0.656	0.0671	0.519 (0.455–0.592)	< 0.001	− 0.238	0.0704	0.788 (0.687–0.905)	< 0.001
Diagnosed by screening (Ref: no)	Yes	− 1.43	0.138	0.24 (0.183–0.314)	< 0.001	− 0.441	0.144	0.643 (0.485–0.853)	0.00219

^#^ Asian-Pacific BMI classification: underweight (BMI < 18.5), normal (18.5 ≥ BMI < 23), overweight (23 ≥ BMI < 25), obese (BMI ≥ 25).

#### Evaluation of potential bias introduced by lung cancer screening

Sensitivity analysis of the Cox model was performed by excluding patients diagnosed by screening or vice versa. XF residency status was not a predictor of OS in the patients diagnosed by screening, but in the group diagnosed by screening, residing in XF was still associated with improved OS (HR = 0.792, 95%CI: 0.688–0.913, *P* = 0.001) (Supplemental Table 2). Simple bias analysis for unmeasured residual confounders was performed to see how the estimates of HR for XR residency status and screening in the Cox model would change by adding on an unmeasured confounder with HR of 0.7, 0.8 or 0.9 (Supplemental Tables 3 and 4). In most situations, patients who were diagnosed by screening or were residing in XF had improved OS in spite of the presence of a favorable unmeasured confounder.

**Figure 3:**
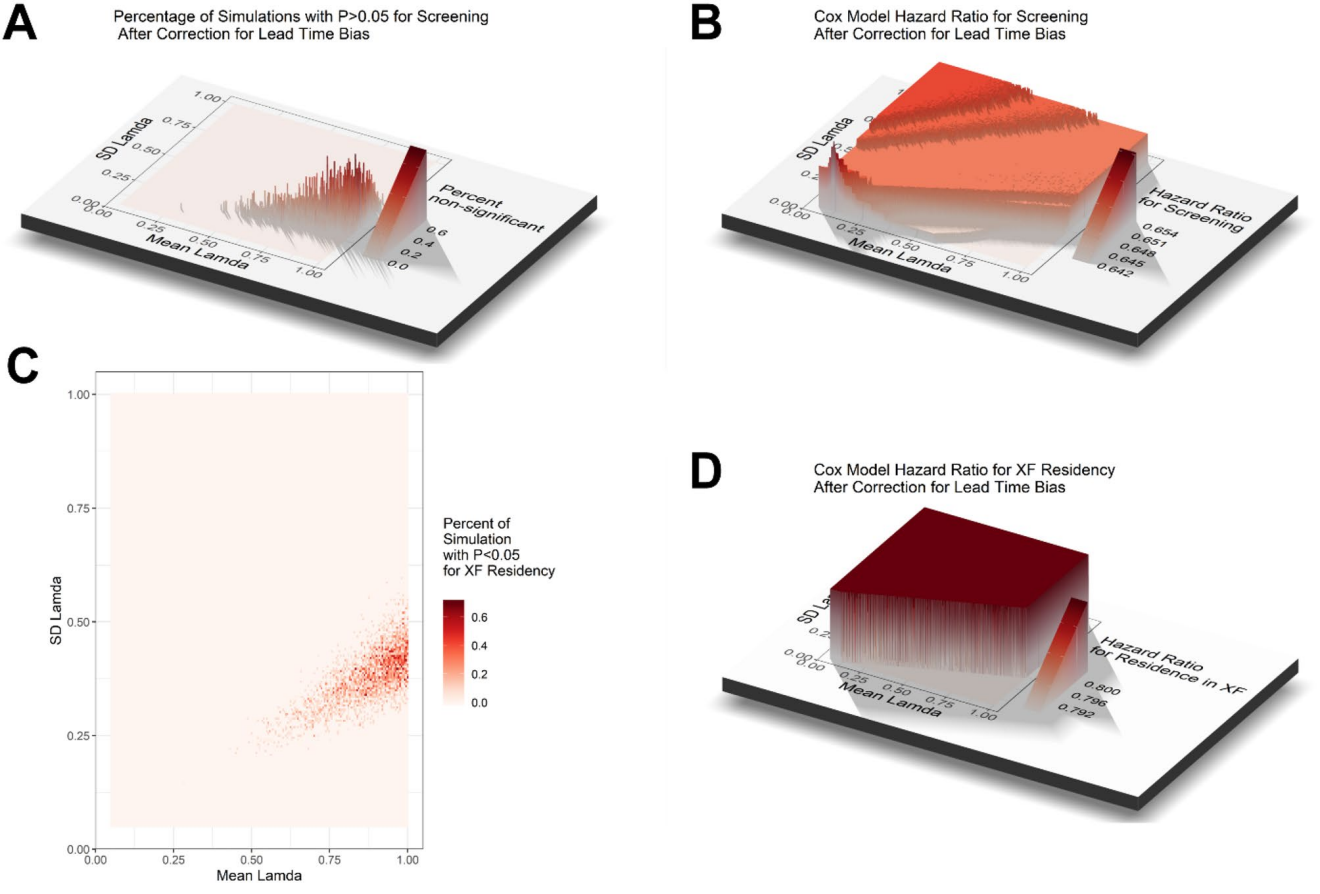
Monte Carlo simulation of lead time correction for overall survival. (**A**) The percentage of simulations with P values for screening in the Cox regression model that changed to > 0.05 after correction for lead time was plotted in 3 dimensions against the mean and standard deviation (SD) of Lamda (λ). (**B**) The hazard ratio (HR) for screening in the Cox regression model after correction for lead time was plotted in 3 dimensions against the mean and SD of λ. (**C**) The percentage of simulations with P values for XF residency status in the Cox regression model that changed to > 0.05 after correction for lead time was presented as a heat map of the mean and SD of λ. (**D**) The HR for XF residency status in the Cox regression model after correction for lead time was plotted in 3 dimensions against the mean and SD of λ. The color keys are shown to the right of each plot.

To explore the potential impact of lead time bias introduced by lung cancer screening on OS of lung cancer patients, we attempted to correct for lead time bias in two ways. First, we tried to subtract an estimated mean lead time from the survival time of patients diagnosed by screening. The mean lead time was estimated to be 3.4 months for OS of stages one and two lung cancer and ≤ 1 month for stages three and four^20^. By subtracting 100 days if stage 1 or 2 and subtracting 30 days if stage 3 or 4 from the survival time of patients diagnosed by screening, the same multivariate Cox regression model in Table 2 using the corrected dataset still showed that both XF residency status (HR = 0.789, 95% CI: 0.687–0.906, *P* < 0.001) and screening (HR = 0.677, 95% CI: 0.511–0.898, *P* = 0.007) were associated with improved OS, and this method of correcting for lead time did not nullify the survival advantage. Second, we tried to estimate lead time using the formula of Duffy et al.: E(s) = (1−e^(-λ t)^)/ λ, where λ is the a rate of transition to symptomatic disease (i.e., mean sojourn time = 1/λ), assuming an exponential distribution of the sojourn time (8). We corrected for lead time by subtracting E(s) from the observed survival time or time to last follow-up of patients diagnosed by screening. E(s) for each screened patient in the cohort was randomly generated using the rnorm function in the R package Compositions with specified mean and standard deviation for λ. For each pair mean and standard deviation for λ, Monte Carlo simulation of the same multivariate Cox model in Table 2 was performed 1000 times to assess the probability of obtaining a *P* > 0.05 for screening or XF residency status. The simulation covered values ranging from 0.05 to 1 for both mean and standard deviation for λ. Lead-time-corrected survival for the patients diagnosed by screening was expected cancers as compared with symptomatic cancers. While the survival of unscreened patient remained the same, lead time correction would lead to a lower survival estimate in the patients diagnosed by screening. By Monte Carlo simulation, we assessed the probability of obtaining a non-significant (*P* ≥ 0.05) HR for screening in the Cox regression model for combinations of mean and standard deviation of λ. The highest probability was < 0.006% (Fig. 3A). The HR for screening ranged from 0.642 to 0.654 (Fig. 3B). Similar results were obtained for the probability of obtaining a non-significant HR for XF residency status (Fig. 3C). The HR for XF residency status ranged from 0.792 to 0.800 (Fig. 3D). Therefore, it was very unlikely or not plausible for the benefits of screening or XF residency status on OS to be completely nullified by lead time bias.

**Figure 4:**
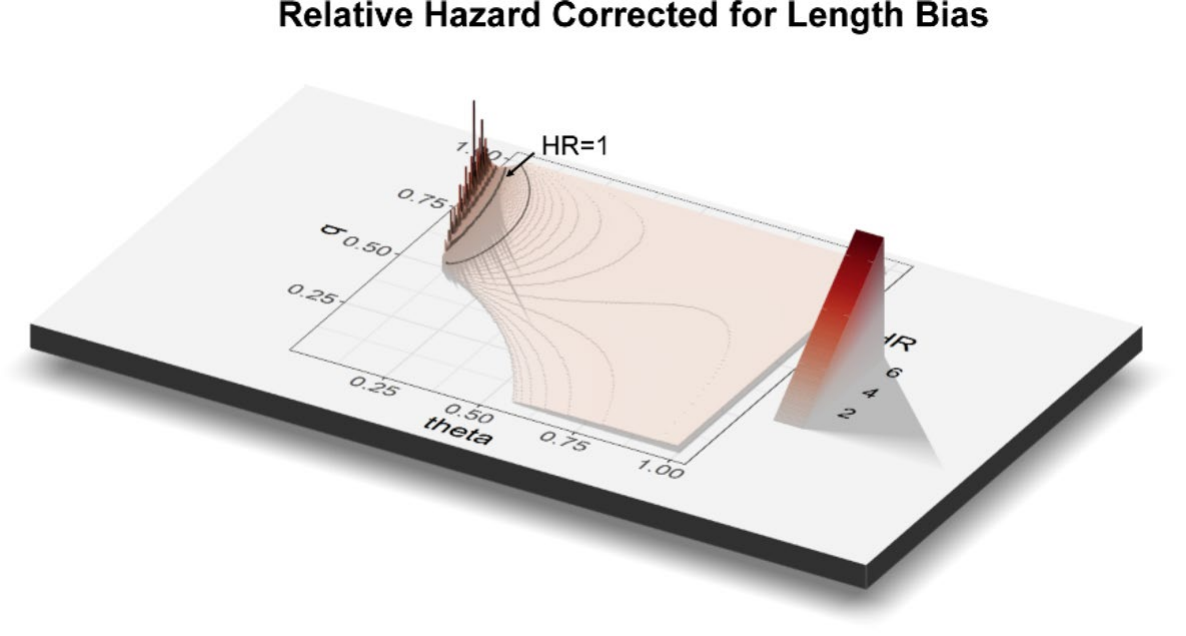
Length bias correction for overall survival. The estimated length bias-corrected relative hazard was plotted in 3 dimensions against q (the complement of the size of the group causing length bias and theta (θ) (the relative rate of screen detection and fatality in the length-bias group). The blank area was due to lack of real solution for the formula for the specific combinations of q and θ. The color key is shown to the right of the plot.

To assess the potential impact of length bias, we used the formula of Duffy et al. to estimate the length bias-corrected relative hazard^23^. We calculated the corrected results for q (the complement of the size of the group causing length bias) ranging from 0 to 1 and θ (the relative rate of screen detection and fatality in the length-bias group) also ranging from 0 to 1. The probability of 5-year case fatality for symptomatic tumors (p1) was 1829/3148 = 0.58, that probability for screen-detected tumors (p2) was 57/270 = 0.21, and the observed probability of diagnosis by screening (p3) was 270/3415 = 0.079. The corrected relative hazard for combinations of q and θ that had real solutions from the formulae were plotted (Fig. 4), and analyses showed that in order for the length bias to account completely for the survival benefit (i.e., corrected relative hazard = 1), the length-bias group would need to be 8 times more likely to be diagnosed by screening and 8 times less likely to cause death.Since the mean sojourn time was 2.24 years for lung cancer (Mayo Lung Project)^27^, it was highly unlikely that length time bias could account for the entire difference in survival associated with screening.

**Table 3:** Shared frailty models for overall survival.

Factor	Level	Diagnosed not by screening				Diagnosed by screening			
		beta	se	HR (95% CI)	p	beta	se	HR (95% CI)	p
Age (Ref: ≤ 65 years)	> 65 years	0.134	0.0542	1.14 (1.03–1.27)	0.0134	0.629	0.46	1.88 (0.762–4.62)	0.172
Sex (Ref: female)	Male	0.19	0.0721	1.21 (1.05–1.39)	0.00841	0.293	0.416	1.34 (0.593–3.03)	0.481
Ethnicity (Ref: non-Han)	Han	0.238	0.0908	1.27 (1.06–1.52)	0.00876	− 1.54	1.1	0.214 (0.0246–1.86)	0.162
TNM stage (Ref: stage I)	Stage II	0.996	0.184	2.71 (1.89–3.88)	< 0.001	− 0.0168	0.622	0.983 (0.29–3.33)	0.978
	Stage III	1.41	0.162	4.1 (2.96–5.6)	< 0.001	0.923	0.471	2.52 (0.999–6.33)	0.05
	Stage IV	1.99	0.299	7.32 (4.08–13.2)	< 0.001	− 0.4	1.02	0.67 (0.0909–4.94)	0.695
Cancer histology (Ref: adenocarcinoma)	Others	0.135	0.071	1.14 (0.996–1.32)	0.0572	− 0.291	0.433	0.748 (0.32–1.75)	0.502
	Small cell	0.1	0.0802	1.11 (0.945–1.29)	0.212	1.21	0.62	3.35 (0.995–11.3)	0.051
	Squamous cell	0.004	0.0703	1 (0.875–1.15)	0.95	0.411	0.605	1.51 (0.461–4.94)	0.497
BMI^#^ (Ref: normal)	Obese	− 0.174	0.0708	0.84 (0.732–0.966)	0.014	0.291	0.361	1.34 (0.66–2.71)	0.42
	Overweight	0.11	0.073	1.12 (0.967–1.29)	0.132	− 2.14	1.08	0.118 (0.014–0.977)	0.0475
	Underweight	− 0.005	0.0645	0.995 (0.877–1.13)	0.938	− 0.754	0.457	0.47 (0.192–1.15)	0.099
Age-unadjusted CCI (Ref: ≤ 3)	> 3	− 0.231	0.258	0.794 (0.479–1.32)	0.371	2.31	0.979	10.1 (1.48–69)	0.0183
KPS (Ref: ≤ 70)	> 70	− 0.222	0.0704	0.801 (0.698–0.919)	0.00161	NA	0	NA (NA-NA)	NA
Smoking (Ref: no)	Yes	0.111	0.0623	1.12 (0.989–1.26)	0.0748	− 0.169	0.404	0.845 (0.383–1.86)	0.676
Surgery (Ref: no)	Yes	− 0.71	0.0794	0.492 (0.421–0.575)	< 0.001	− 1.67	0.373	0.188 (0.091–0.393)	< 0.001
Chemotherapy (Ref: no)	Yes	− 0.42	0.0569	0.657 (0.587–0.734)	< 0.001	− 0.143	0.362	0.867 (0.427–1.76)	0.693
Radiotherapy (Ref: no)	Yes	− 0.256	0.0691	0.774 (0.676–0.887)	< 0.001	− 0.121	0.446	0.886 (0.369–2.12)	0.786
Targeted therapy (Ref: no)	Yes	− 0.315	0.0843	0.73 (0.619–0.861)	< 0.001	0.00522	0.525	1.01 (0.359–2.81)	0.992
XF resident (Ref: no)	Yes	Frailty			0.0021	Frailty			NA

#### Evaluation of a latent common group effect by shared frailty modeling

A shared frailty random effects model may account for the regional heterogeneity in survival data^28^. After separating the cohort into subcohorts of patients diagnosed by screening or not, we used shared frailty models to examine for the presence of significant frailty term for covariates. The frailty term for XF residency modified the hazard multiplicatively and assigns each patient in XF or non-XF region the same level of frailty. The result was compared with the Cox models without a frailty term to determine the impact of clustering by regions of residence. Models using gamma, gaussian or t distributions all yielded similar results, and only results using gamma distribution were shown in Table 3. There was no frailty associated with residence in XF in patients diagnosed by screening, but there was significant (*P* = 0.0021) frailty associated with XF residency status in patients diagnosed not by screening. In the shared frailty models for patients diagnosed by screening or those diagnosed by screening, patients in a cluster (residing in XF or residing elsewhere in Yunnan) were assumed to share the same unmeasured/unobserved risk factor (frailty). Therefore, while there was no significant unmeasured heterogeneity between the clusters (XF vs non-XF), there was significant unmeasured heterogeneity between XF vs. non-XF residents that cannot be explained by observed covariates alone.

## Discussion

The burden of lung cancer worldwide is increasing from smoking and air pollution^29^. In countries like India, China and regions that are still using biomass fuels, females get lung cancer from indoor air pollution due to cooking fumes and poor ventilation^30,31^. In contrast to decreasing lung cancer incidence rates in Western countries, lung cancer incidence rates are still rising in China, other Asian countries in and Africa^30,32^^.^ The 5-year lung cancer survival rates was 13% in 1975–1977 and 1984–1986 but did not improve by much to only 16% for 1999–2005^33^. In 2015, the cancer data statistics from China showed that the lung cancer incidence and mortality rate were both the highest among cancers for both sexes^9^. The XF region in Yunnan Province (a province in southwestern China) is a high-incidence area for lung cancer due to air pollution from burning of bituminous coal and geographic factors. Efforts for lung cancer screening, raising public awareness and reduction of environmental exposure (e.g., vented stoves) have been ongoing to try to improve lung cancer mortality.

The current study used institutional data from a regional cancer center to examine lung cancer survival in Yunnan. This retrospective cohort consisted of newly diagnosed cases of lung cancer in Yunnan Cancer Hospital from January 1st, 2012 to July 10th, 2015, a cohort more recent when compared to other studies^33^. The relatively low male: female ratio and the high percentage of adenocarcinoma among cancer histopathological types perhaps reflected the dominance of coal-related air pollution in lung carcinogenesis in XF. We found that the XF lung cancer patients were more likely to be diagnosed by screening than non-XF patients. Both the XF and non-XF patients had 5-year survival rates that were markedly higher than reported about a decade ago^33^. They were younger, in better general health conditions, in earlier malignancy stages, and more likely to undergo surgery for treatment than non-XF patients. These observations were likely due to prior years of effort in lung cancer screening and raising public awareness about lung cancer.

XF lung cancer patients appeared to have better OS than non-XF patients. Kaplan–Meier analysis of OS showed that XF patients survive longer (difference in median survival > 3.5 years) than non-XF patients. This finding was confirmed by univariate proportional hazard analysis, i.e., 42% reduction in hazard for XF patients. Multivariate analysis of OS using Cox proportional hazard modeling showed that XF patients were less likely to die than non-XF patients after adjusting for demographic, clinicopathological, and treatment factors. The covariates in the Cox model included the top 12 most important factors identified by random forest survival analysis. In addition to residence in XF being a significant factor, the results from the Cox model for the entire cohort also showed that increased age, male sex, non-Han ethnic minority and increased stage were adverse risk factors and that being overweight, increased Karnofsky performance score, surgery, chemotherapy, radiotherapy and tyrosine kinase inhibitor targeted therapy were beneficial risk factors.

Lung cancer screening has been supported by the findings of the Dutch–Belgian NELSON (Nederlands-Leuven Longkanker Screenings Network) Randomized Lung Cancer Screening Trial^34^ and the National Lung Screening Trial (NLST)^35^. NELSON was the largest European lung cancer CT screening trial, and it confirmed the findings of the NLST. These trials and real world implementation in Taiwan^13,36^ showed that screening can increase the proportion of lung cancer diagnosed in early stages with curative possibility. Lung cancer screening policies vary from country to country and region to region; outside the setting of a formal clinical trial, there is little data to show that real-world screening effort (governmental or civilian) do make a difference in survival in general. Moreover, the patient’s decision to undergo lung cancer screening is likely to be influenced by availability, out-of-pocket/financial cost and self-awareness of lung cancer risk^37^, which will vary region-to-region due to local prevalence of lung cancer, presence of risk factors, public awareness, and promotion of lung cancer screening (both governmental, health care industry, or philanthropic).

Cancer screening introduces biases that complicate interpretations of survival analysis. Ongoing lung cancer screening effort has focused on high risk regions that included XF, including grants funded by the government^38,39^, provincial^40^ and local^41^ hospital efforts, and collaboration among governmental agencies, private foundations and philanthropic groups^42^. The Chinese National Lung Cancer Screening Guidelines specifically mentioned the high risk group in XF^43,44^. It was quite obvious that lung cancer screening effort had focused on the XF region since 16.8% of lung cancer cases in XF patients were diagnosed through screening compared with 5.0% in non-XF patients. Length bias is associated with slow growing tumors with a long presymptomatic screen-detectable duration, but lung cancers generally do not fit this profile of clinical progression, and our analysis using the method by Duffy et al.^23^ showed that it was not plausible for length bias to account for all the survival benefits of screening in our data set. Lead time bias, however, is an important issue for in analysis of OS, which is defined as the time duration from diagnosis to death, because the lead time would artificially lengthen OS in screen-diagnosed cases. The mean lead time has been estimated to be 3.4 months for OS of early stage (I or II) lung cancer patients, and only ≤ 1 month for advanced stage (III or IV) lung cancer^20^. Given the large difference in median survival between XF and non-XF patients (> 3.5 years) and our simulation of lead time bias, lead time bias could not nullify the survival advantage of screening in our data set. To examine the impact of lead time bias in survival analysis, sensitivity analysis by dividing the cohort into those that were diagnosed by screening or not showed that residence in XF was an independent factor associated with improved survival in XF residents but no longer a significant factor for OS in the subcohort diagnosed by screening.

In a frailty model, the random component accounts for association unknown factors and unobserved heterogeneity^45,46^. A frailty is an unobserved random factor that modifies multiplicatively the risk of event occurrence of a cluster of individuals. In our analysis (Table 3), the lung cancer patients were clustered within their region of residence and possible correlation between patients within XF or non-XF were modeled with a shared frailty model, and we found that a significant unobserved random factor related to the region of residence was present in patients diagnosed not by screening but not in those diagnosed by screening. Therefore, other than screening, there remained important variations in lung cancer outcomes linked to region of residence. These unobserved factors potentially include socio-economic status, genetic prognostic factors or biological markers of prognosis, and variations in relation to local patterns of oncology follow up and care delivery^47–49^, which may be influenced by increased governmental investment in health care resources^50^.

A limitation of our study was that the data came from a single institution. Even though Yunnan Cancer Hospital is the only tertiary referral center for cancer care in Yunnan province, not all lung cancer patients in XF or non-XF regions would have sought care in this hospital. Although unsuccessful telephone follow up may possibly be associated with worse prognosis, the rates of unsuccessful follow up were essentially the same for XF and non-XF groups. Therefore, its impact would equally affect both the XF and non-XF groups and not bias one group against another. The sample size of lung cancer patients diagnosed by screening was relatively small. The patients who were diagnosed by screening in our study cohort were a mixture of patients diagnosed in real life clinical practice and participants in various lung cancer screening programs which might have participation rates of the target population to be screened that varied from 31.91% to 84.5%^51,52^. Given the retrospective nature of our study, errors in data collection might have occurred although care was taken to independently confirm the data wherever possible (e.g., verification of region of residence using geocode and map plotting). Efforts are in progress to discourage smoking, decrease air pollution and continue cancer screening, and these will affect the generalizability and applicability of our results over time.

In conclusion, the improved survival for lung cancer patients in the XF region is the combined effect of higher percentage of patients diagnosed by screening and unobserved factors associated with the region of residence. The magnitude of the survival advantage could not be fully accounted for by lead time bias introduced by screening. In patients diagnosed by screening, the lung cancer was likely to be diagnosed at an early stage, and surgery was the most important and influential factor for survival. This is perhaps the first report that have shown that lung cancer screening effort has produced a beneficial effect on survival of lung cancer patients in Yunnan. Long term prospective studies are needed to confirm our findings. Our results would justify further promotion of cancer screening, early diagnosis and treatment in an efficient, effective manner for this province and other regions, particularly those that are underserved.

## Supplementary information

Supplementary file1

Supplementary file2

Supplementary file3
