# Supplementary material for: Association of cancer screening and residing in a coal-polluted East Asian region with overall survival of lung cancer patients: a retrospective cohort study
Source: Sci Rep. 2020 Oct 15;10:17432. doi: 10.1038/s41598-020-74082-0 (PMC7566617; doi:10.1038/s41598-020-74082-0)
Supplement: Supplementary file 1 — Supplementary file1 [file 41598_2020_74082_MOESM1_ESM.docx]

**Supplemental Material**

**Title:** Association of cancer screening and residing in a coal-polluted East Asian region with overall survival of lung cancer patients: a retrospective cohort study

Runxiang Yang,^1,^* Ming He,^1^ Dongmei Wang,^1^ Rongrong Ye,^1^ Lu Li^2^, Rouyu Deng,^1^ Mohsin Shah^3^, Sai-Ching Jim Yeung^4^

^1^The Second Department of Medical Oncology, Yunnan Cancer Hospital, Kunming, Yunnan, The People’s Republic of China

^2^Department of Oncology, Sun Yat-sen University Cancer Center, Guangzhou, Guangdong, The People’s Republic of China

^3^Center for Clinical Epidemiology and Biostatistics, Perelman School of Medicine, University of Pennsylvania, Philadelphia, Pennsylvania, USA

^4^Department of Emergency Medicine, The University of Texas MD Anderson Cancer Center, Houston, Texas, USA

*** Corresponding author**

Runxiang Yang

Email: [13888876721@163.com](mailto:13888876721@163.com), Tel: 86-1388876721

**Word Count:** 3693

**Keywords:** Lung cancer, Xuanwei-Fuyuan region, air pollution, Yunnan, survival, cancer screening

**Running Head:** Improved survival of lung cancer patients in East Asia

**Supplemental Figure Legends**

Supplemental Figure 1: Patient cohort.

The flow chart indicates the number of patients in the process of assembling the study cohort.

Supplemental Figure 2: Geographic distribution of the analyzed cases.

Red dots represent cases from Xuanwei and Fuyuan counties, and blue dots, the rest of Yunnan Province. The color dots are semi-transparent, and multiple overlapping dots will result in color saturation.

Supplemental Table 1. Ratio of XF residents to non-XF residents in the cohort each year over the study period.

Year of diagnosis 2012 2013 2014 2015

XF residents 176 185 137 192

Non-XF residents 637 729 633 716

XF:non-XF ratio 1:3.6 1:3.9 1:4.6 1:3.7

Supplemental Table 2. Multivariate Cox proportional hazard models of overall survival for patients diagnosed by screening or not.

|  | | Diagnosed by Screening | | | | No Screening | | | |
| --- | --- | --- | --- | --- | --- | --- | --- | --- | --- |
| Factor | Level | beta | se | HR | p | beta | se | HR | p |
| Age (Ref: ≤65 years) | >65 years | 0.555 | 0.468 | 1.74 (0.695-4.36) | 0.236 | 0.131 | 0.0542 | 1.14 (1.03-1.27) | 0.0157 |
| Sex (Ref: female) | male | 0.304 | 0.417 | 1.36 (0.598-3.07) | 0.466 | 0.188 | 0.0721 | 1.21 (1.05-1.39) | 0.00912 |
| Ethnicity (Ref: non-Han) | Han | -1.44 | 1.1 | 0.237 (0.0274-2.04) | 0.191 | 0.234 | 0.0908 | 1.26 (1.06-1.51) | 0.00996 |
| TNM stage (Ref: stage I) | stage II | 0.0018 | 0.628 | 1 (0.293-3.43) | 0.998 | 0.995 | 0.184 | 2.7 (1.89-3.88) | <0.001 |
|  | stage III | 0.966 | 0.475 | 2.63 (1.04-6.67) | 0.042 | 1.4 | 0.162 | 4.06 (2.96-5.59) | 0 |
|  | stage IV | -0.453 | 1.02 | 0.636 (0.0864-4.68) | 0.657 | 1.99 | 0.299 | 7.32 (4.08-13.2) | <0.001 |
| Cancer histology  (Ref: adenocarcinoma) | others | -0.261 | 0.44 | 0.77 (0.325-1.82) | 0.553 | 0.136 | 0.0709 | 1.15 (0.997-1.32) | 0.0551 |
|  | small cell | 1.25 | 0.614 | 3.49 (1.05-11.6) | 0.0418 | 0.0988 | 0.0802 | 1.1 (0.943-1.29) | 0.218 |
|  | squamous cell | 0.317 | 0.619 | 1.37 (0.408-4.62) | 0.609 | 0.00133 | 0.0703 | 1 (0.872-1.15) | 0.985 |
| BMI^#^ (Ref: normal) | obese | 0.311 | 0.361 | 1.36 (0.673-2.77) | 0.389 | -0.174 | 0.0708 | 0.84 (0.731-0.965) | 0.014 |
|  | overweight | -2.12 | 1.08 | 0.12 (0.0144-1) | 0.0497 | 0.11 | 0.073 | 1.12 (0.967-1.29) | 0.132 |
|  | underweight | -0.75 | 0.454 | 0.472 (0.194-1.15) | 0.0985 | -0.00417 | 0.0645 | 0.996 (0.878-1.13) | 0.948 |
| Age-unadjusted CCI (Ref: ≤3) | >3 | 2.39 | 0.984 | 10.9 (1.58-75) | 0.0151 | -0.231 | 0.258 | 0.794 (0.478-1.32) | 0.371 |
| KPS (Ref: ≤70) | >70 | NA | 0 | NA | NA | -0.222 | 0.0704 | 0.801 (0.698-0.919) | 0.00161 |
| Smoking (Ref: no) | yes | -0.122 | 0.405 | 0.885 (0.401-1.96) | 0.763 | 0.114 | 0.0623 | 1.12 (0.991-1.27) | 0.0673 |
| Surgery (Ref: no) | yes | -1.62 | 0.378 | 0.198 (0.0947-0.416) | <0.001 | -0.704 | 0.0795 | 0.495 (0.423-0.578) | <0.001 |
| Chemotherapy (Ref: no) | yes | -0.198 | 0.368 | 0.82 (0.399-1.69) | 0.591 | -0.421 | 0.0569 | 0.656 (0.587-0.734) | <0.001 |
| Radiotherapy (Ref: no) | yes | -0.0915 | 0.438 | 0.913 (0.387-2.15) | 0.835 | -0.255 | 0.0691 | 0.775 (0.677-0.888) | <0.001 |
| Targeted therapy (Ref: no) | yes | 0.00148 | 0.523 | 1 (0.36-2.79) | 0.998 | -0.318 | 0.0843 | 0.728 (0.617-0.858) | <0.001 |
| XF resident (Ref: no) | yes | -0.262 | 0.315 | 0.77 (0.415-1.43) | 0.406 | -0.233 | 0.0723 | 0.792 (0.688-0.913) | 0.00127 |

^#^ Asian-Pacific BMI classification: underweight (BMI<18.5), normal (18.5 ≥BMI<23), overweight (23≥BMI<25), obese (BMI≥25).

Supplemental Table 3. Simple bias analysis of screening by sensitivity analysis for an unmeasured residual categorical confounder

| Prevalence of unmeasured confounder (%) | | Hazard ratio adjusted for unmeasured confounder (95%CI) | | |
| --- | --- | --- | --- | --- |
| Group diagnosed not by screening | Group diagnosed by screening | Unmeasured confounder HR=0.7 | Unmeasured confounder HR=0.8 | Unmeasured confounder HR=0.9 |
| 0 | 0 | 0.643 (0.485-0.853) | 0.643 (0.485-0.853) | 0.643 (0.485-0.853) |
|  | 20 | 0.605 (0.456-0.802) | 0.618 (0.466-0.819) | 0.605 (0.456-0.802) |
|  | 40 | 0.566 (0.427-0.751) | 0.592 (0.446-0.785) | 0.566 (0.427-0.751) |
|  | 60 | 0.528 (0.398-0.700) | 0.566 (0.427-0.751) | 0.528 (0.398-0.700) |
|  | 80 | 0.489 (0.369-0.648) | 0.540 (0.408-0.717) | 0.489 (0.369-0.648) |
|  | 100 | 0.450 (0.340-0.597) | 0.515 (0.388-0.683) | 0.450 (0.340-0.597) |
| 20 | 0 | 0.685 (0.516-0.908) | 0.670 (0.505-0.889) | 0.657 (0.495-0.871) |
|  | 20 | 0.643 (0.485-0.853) | 0.643 (0.485-0.853) | 0.643 (0.485-0.853) |
|  | 40 | 0.602 (0.454-0.799) | 0.617 (0.465-0.818) | 0.630 (0.475-0.836) |
|  | 60 | 0.561 (0.423-0.744) | 0.590 (0.445-0.782) | 0.617 (0.465-0.818) |
|  | 80 | 0.520 (0.392-0.690) | 0.563 (0.425-0.747) | 0.604 (0.456-0.801) |
|  | 100 | 0.479 (0.361-0.635) | 0.536 (0.404-0.711) | 0.591 (0.446-0.784) |
| 40 | 0 | 0.731 (0.551-0.970) | 0.699 (0.527-0.927) | 0.670 (0.505-0.889) |
|  | 20 | 0.687 (0.518-0.911) | 0.671 (0.506-0.890) | 0.657 (0.495-0.871) |
|  | 40 | 0.643 (0.485-0.853) | 0.643 (0.485-0.853) | 0.643 (0.485-0.853) |
|  | 60 | 0.600 (0.452-0.795) | 0.615 (0.464-0.816) | 0.630 (0.475-0.835) |
|  | 80 | 0.556 (0.419-0.737) | 0.587 (0.443-0.779) | 0.617 (0.465-0.818) |
|  | 100 | 0.512 (0.386-0.679) | 0.560 (0.4220.742) | 0.603 (0.455-0.800) |
| 60 | 0 | **0.785 (0.592-1.041)** | 0.731 (0.551-0.970) | 0.685 (0.516-0.908) |
|  | 20 | 0.738 (0.556-0.978) | 0.702 (0.529-0.931) | 0.671 (0.506-0.890) |
|  | 40 | 0.691 (0.521-0.916) | 0.673 (0.507-0.892) | 0.657 (0.496-0.871) |
|  | 60 | 0.643 (0.485-0.853) | 0.643 (0.485-0.853) | 0.643 (0.485-0.853) |
|  | 80 | 0.596 (0.450-0.791) | 0.614 (0.463-0.814) | 0.630 (0.475-0.835) |
|  | 100 | 0.549 (0.414-0.728) | 0.585 (0.441-0.776) | 0.616 (0.465-0.817) |
| 80 | 0 | **0.847 (0.638-1.123)** | **0.766 (0.578-1.016)** | 0.699 (0.527-0.927) |
|  | 20 | **0.796 (0.600-1.055)** | 0.735 (0.555-0.975) | 0.685 (0.517-0.909) |
|  | 40 | 0.745 (0.562-0.988) | 0.705 (0.531-0.934) | 0.671 (0.506-0.890) |
|  | 60 | 0.694 (0.524-0.921) | 0.674 (0.508-0.894) | 0.657 (0.496-0.872) |
|  | 80 | 0.643 (0.485-0.853) | 0.643 (0.485-0.853) | 0.643 (0.485-0.853) |
|  | 100 | 0.593 (0.447-0.786) | 0.613 (0.462-0.813) | 0.629 (0.475-0.835) |
| 100 | 0 | **0.919 (0.693-1.219)** | **0.804 (0.607-1.067)** | 0.715 (0.539-0.948) |
|  | 20 | **0.864 (0.652-1.146)** | **0.772 (0.582-1.024)** | 0.701 (0.528-0.929) |
|  | 40 | **0.809 (0.610-1.073)** | 0.740 (0.558-0.981) | 0.686 (0.518-0.910) |
|  | 60 | 0.754 (0.568-0.999) | 0.708 (0.534-0.939) | 0.672 (0.507-0.891) |
|  | 80 | 0.699 (0.527-0.926) | 0.676 (0.510-0.896) | 0.658 (0.496-0.872) |
|  | 100 | 0.643 (0.485-0.853) | 0.643 (0.485-0.853) | 0.643 (0.485-0.853) |

Bold type: P>0.05

Supplemental Table 4. Simple bias analysis of residence in Xuanwei-Fuyuan area by sensitivity analysis for an unmeasured residual categorical confounder

| Prevalence of unmeasured confounder (%) | | Hazard ratio adjusted for unmeasured confounder (95%CI) | | |
| --- | --- | --- | --- | --- |
| Group residing outside Xuanwei-Fuyuan area | Group residing inside Xuanwei-Fuyuan area | Unmeasured confounder HR=0.7 | Unmeasured confounder HR=0.8 | Unmeasured confounder HR=0.9 |
| 0 | 0 | 0.789 (0.687-0.905) | 0.789 (0.687-0.905) | 0.789 (0.687-0.905) |
|  | 20 | 0.741 (0.646-0.851) | 0.757 (0.659-0.869) | 0.773 (0.673-0.887) |
|  | 40 | 0.694 (0.604-0.797) | 0.726 (0.632-0.833) | 0.757 (0.659-0.869) |
|  | 60 | 0.647 (0.563-0.742) | 0.694 (0.604-0.797) | 0.741 (0.646-0.851) |
|  | 80 | 0.599 (0.522-0.688) | 0.662 (0.577-0.760) | 0.726 (0.632-0.833) |
|  | 100 | 0.552 (0.481-0.634) | 0.631 (0.550-0.724) | 0.710 (0.618-0.815) |
| 20 | 0 | 0.839 (0.731-0.963) | 0.821 (0.716-0.943) | 0.805 (0.701-0.924) |
|  | 20 | 0.789 (0.687-0.905) | 0.789 (0.687-0.905) | 0.789 (0.687-0.905) |
|  | 40 | 0.738 (0.643-0.848) | 0.756 (0.658-0.868) | 0.773 (0.673-0.887) |
|  | 60 | 0.688 (0.599-0.790) | 0.723 (0.630-0.830) | 0.756 (0.659-0.868) |
|  | 80 | 0.638 (0.555-0.732) | 0.690 (0.630-0.830) | 0.740 (0.645-0.850) |
|  | 100 | 0.587 (0.512-0.674) | 0.657 (0.572-0.754) | 0.724 (0.631-0.831) |
| 40 | 0 | **0.896 (0.781-1.029)** | 0.857 (0.747-0.984) | 0.821 (0.716-0.943) |
|  | 20 | **0.842 (0.781-1.029)** | 0.823 (0.717-0.945) | 0.805 (0.701-0.924) |
|  | 40 | 0.789 (0.687-0.905) | 0.789 (0.687-0.905) | 0.789 (0.687-0.905) |
|  | 60 | 0.735 (0.640-0.844) | 0.754 (0.657-0.866) | 0.772 (0.673-0.886) |
|  | 80 | 0.681 (0.593-0.782) | 0.720 (0.627-0.827) | 0.756 (0.658-0.868) |
|  | 100 | 0.627 (0.546-0.720) | 0.686 (0.597-0.787) | 0.739 (0.644-0.849) |
| 60 | 0 | **0.962 (0.838-1.104)** | **0.896 (0.781-1.029)** | 0.839 (0.731-0.963) |
|  | 20 | **0.904 (0.787-1.038)** | 0.860 (0.749-0.988) | 0.822 (0.716-0.944) |
|  | 40 | 0.846 (0.737-0.972) | 0.824 (0.718-0.946) | 0.805 (0.702-0.925) |
|  | 60 | 0.789 (0.687-0.905) | 0.789 (0.687-0.905) | 0.772 (0.687-0.905) |
|  | 80 | 0.731 (0.637-0.839) | 0.753 (0.656-0.864) | 0.772 (0.672-0.886) |
|  | 100 | 0.673 (0.586-0.773) | 0.717 (0.624-0.823) | 0.755 (0.658-0.867) |
| 80 | 0 | **1.038 (0.904-1.191)** | **0.939 (0.818-1.078)** | 0.857 (0.747-0.984) |
|  | 20 | **0.975 (0.850-1.120)** | **0.901 (0.785-1.035)** | 0.840 (0.732-0.964) |
|  | 40 | **0.913 (0.795-1.048)** | 0.864 (0.752-0.992) | 0.823 (0.717-0.945) |
|  | 60 | 0.851 (0.741-0.977) | 0.826 (0.720-0.948) | 0.806 (0.702-0.925) |
|  | 80 | 0.789 (0.687-0.905) | 0.789 (0.687-0.905) | 0.789 (0.687-0.905) |
|  | 100 | 0.726 (0.633-0.834) | 0.789 (0.687-0.905) | 0.771 (0.672-0.886) |
| 100 | 0 | **1.127 (0.981-1.293)** | **0.986 (0.859-1.132)** | **0.876 (0.763-1.006)** |
|  | 20 | **1.059 (0.922-1.216)** | **0.946 (0.824-1.086)** | 0.859 (0.748-0.986) |
|  | 40 | **0.991 (0.864-1.138)** | **0.907 (0.790-1.041)** | 0.841 (0.733-0.966) |
|  | 60 | **0.924 (0.805-1.061)** | 0.867 (0.756-0.996) | 0.824 (0.717-0.946) |
|  | 80 | 0.856 (0.746-0.983) | 0.828 (0.721-0.951) | 0.806 (0.702-0.925) |
|  | 100 | 0.789 (0.687-0.905) | 0.789 (0.687-0.905) | 0.789 (0.687-0.905) |

Bold type: P>0.05
