# Supplementary figures and images for: Association of cancer screening and residing in a coal-polluted East Asian region with overall survival of lung cancer patients: a retrospective cohort study
Source: Sci Rep. 2020 Oct 15;10:17432. doi: 10.1038/s41598-020-74082-0 (PMC7566617; doi:10.1038/s41598-020-74082-0)

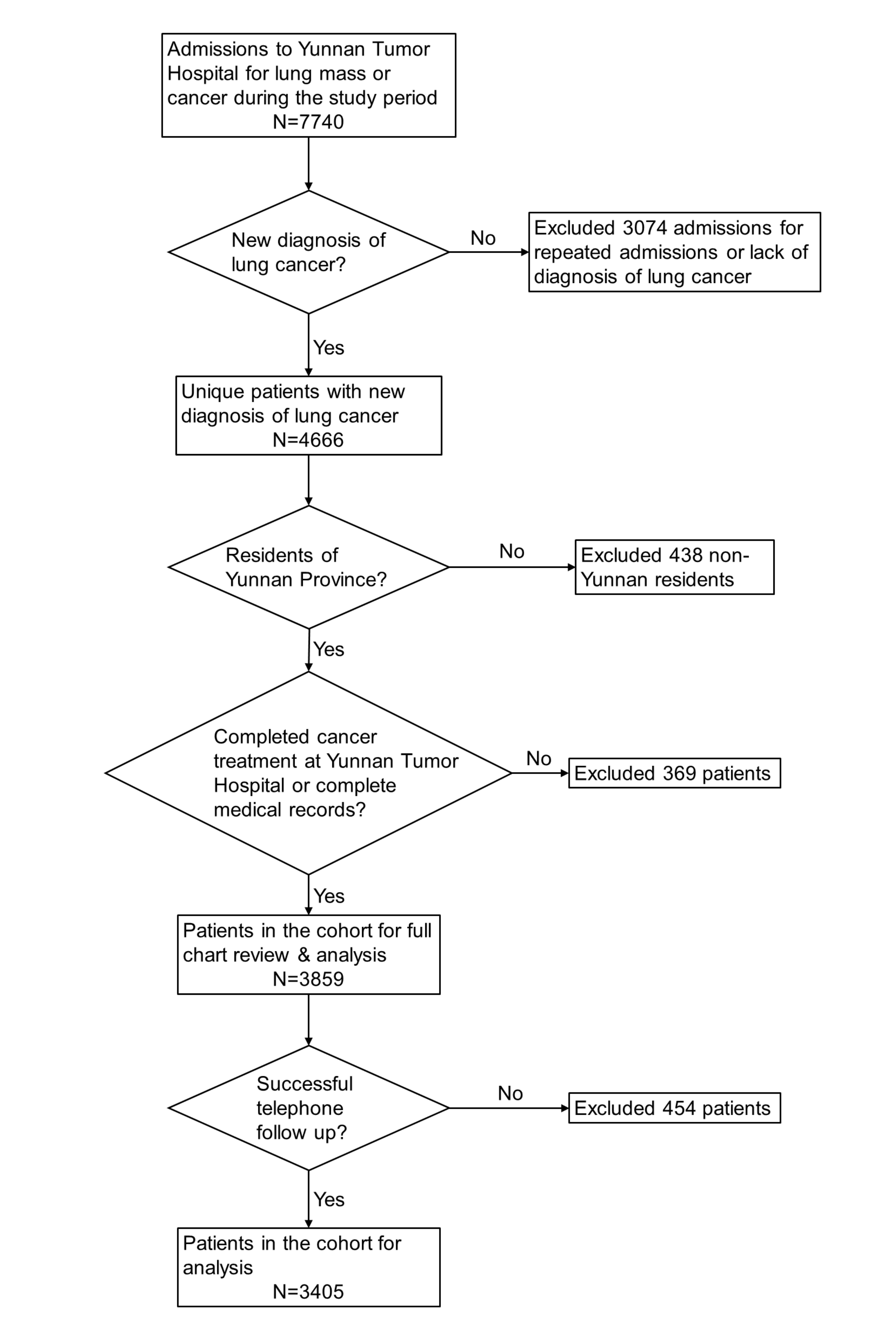

Supplement: Supplementary file 2 — Supplementary file2 [file 41598_2020_74082_MOESM2_ESM.tif]

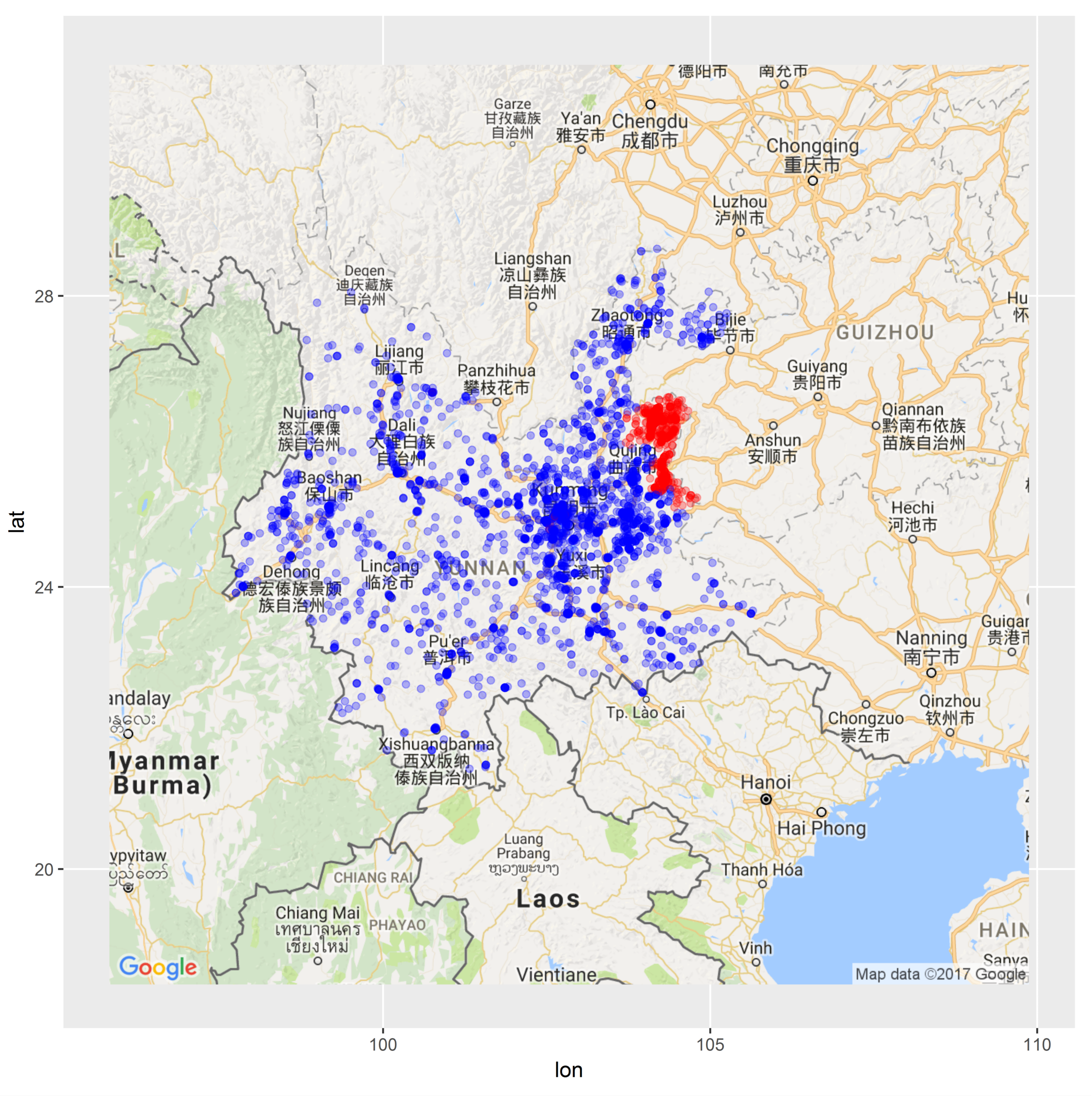

Supplement: Supplementary file 3 — Supplementary file3 [file 41598_2020_74082_MOESM3_ESM.tif]
